# Bacteriological Profile and Antibiotic Sensitivity Pattern of Burn Wounds in a Tertiary Burn Care Center

**DOI:** 10.7759/cureus.110010

**Published:** 2026-05-31

**Authors:** Muhammad Ronuq Hasan, Shariff A Rahman, Kafi Chowdhury, Kawsar Ahmad, Shafia Nasrin, Israt Jahan, Mst Samiha Islam

**Affiliations:** 1 Plastic Surgery, Thengamara Mohila Sabuj Sangha (TMSS) Medical College and Rafatullah Community Hospital, Bogura, BGD; 2 Plastic and Reconstructive Surgery, National Institute of Burn and Plastic Surgery (NIBPS), Dhaka, BGD; 3 Plastic Surgery, Shahid M. Monsur Ali Medical College and Hospital, Dhaka, BGD; 4 Plastic Surgery, National Institute of Burn and Plastic Surgery (NIBPS), Dhaka, BGD; 5 Pediatrics, Bangladesh Navy Hospital (BNS Upasham), Khulna, BGD; 6 Surgery, Thengamara Mohila Sabuj Sangha (TMSS) Medical College and Rafatullah Community Hospital, Bogura, BGD

**Keywords:** antibiogram, antibiotic sensitivity, bacteriological profile, burn, wound infection

## Abstract

Background: Burn wounds provide a favorable environment for opportunistic pathogens, leading to microbial colonization, infection-related complications, prolonged hospitalization, and increased morbidity, particularly in low- and middle-income countries. Understanding the bacteriological profile and antibiotic sensitivity is essential for guiding therapy and curbing inappropriate antibiotic use. Therefore, this study aimed to determine the bacteriological profile of burn wounds and assess antimicrobial sensitivity on the 1st, 5th, and 10th post-burn days in a tertiary burn care center.

Materials and methods: A prospective observational study was conducted at National Institute of Burn and Plastic Surgery (NIBPS), Dhaka, Bangladesh, from July 2022 to December 2023. A total of 110 patients with burns involving 10-50% of total body surface area were enrolled and followed longitudinally. Wound swabs were collected from the same patients on the first, fifth, and 10th post-burn days to assess the early, intermediate, and later phases of bacterial colonization and antimicrobial susceptibility changes over time. Isolates were identified by standard microbiological techniques, and antibiotic susceptibility testing was performed using the CLSI disc diffusion method.

Results: Children under 10 years constituted the largest proportion of cases (43.6%). Flame burns predominated (50%), with the trunk most commonly affected (73.6%). Bacterial growth increased from 36.4% on day 1 to 86.4% by day 5 and remained high on day 10. Gram-negative organisms predominated throughout the study period, with Pseudomonas aeruginosa being the most frequent isolate. None of the tested antibiotics demonstrated consistently high susceptibility. Colistin demonstrated comparatively higher susceptibility than other tested antibiotics, although susceptibility rates remained suboptimal.

Conclusion: Burn wounds showed rapid colonization predominantly by gram-negative organisms, particularly Pseudomonas aeruginosa, with low antibiotic susceptibility across several commonly used agents. The findings highlight the potential importance of continuous microbiological surveillance, burn unit-specific antibiograms, antimicrobial stewardship, and rational antibiotic use in burn care settings.

## Introduction

Burn injury is among the most devastating forms of trauma and remains a major public health concern worldwide [1,2]. Burns cause an estimated 180,000 deaths annually, predominantly in low- and middle-income countries, particularly in the WHO African and South-East Asia regions. Although burn-related mortality has declined in many high-income countries, child burn mortality remains more than seven times higher in low- and middle-income settings. In Bangladesh alone, nearly 173,000 children sustain moderate to severe burns each year, with many survivors experiencing temporary or permanent disability [3].

Chemicals, hot liquids, electricity, and molten metals are common causes of burns, with the extent of damage depending on skin thickness, exposure duration, and heat intensity [4]. Such injuries disrupt the skin barrier, creating a moist, nutrient-rich environment that facilitates bacterial colonization, thereby increasing infection risk and delaying healing [5-7].

Burn wound colonization is a dynamic process involving both gram-positive and gram-negative organisms, including Staphylococcus aureus, Pseudomonas aeruginosa, Acinetobacter baumannii, Escherichia coli, and Klebsiella pneumoniae [2]. While gram-positive organisms remain important, gram-negative bacteria increasingly dominate and often exhibit resistance to broad-spectrum antibiotics. Prolonged antibiotic use also predisposes to fungal infections, and multidrug-resistant or polymicrobial infections are strongly associated with poor outcomes, with sepsis accounting for nearly 73% of post-burn deaths within five days [8].

In burn care units, antibiotic stewardship is essential, and every burn unit should maintain an updated antibiogram to optimize treatment strategies for sepsis [9]. When culture and sensitivity are unavailable, clinicians often initiate broad empirical therapy that covers both gram-positive and gram-negative organisms, with antifungal agents considered in prolonged cases. Although prophylactic antibiotics remain widely practiced [10], the emergence of resistant pathogens, favored by intrinsic microbial mechanisms, prolonged hospital persistence, and cross-transmission, has become a critical concern [11]. This challenge is compounded by injudicious use of systemic and topical broad-spectrum agents, while in many low-resource countries, easy over-the-counter access without a prescription further accelerates antimicrobial misuse and resistance [12,13].

Although several Indian and Middle Eastern studies have profiled burn wound pathogens, prospective multi-day surveillance studies remain scarce in Bangladesh despite hosting one of the world’s largest burn hospitals. This study aimed to evaluate temporal changes in burn wound bacterial colonization and antimicrobial susceptibility patterns during hospitalization to guide empirical therapy and improve patient outcomes.

## Materials and methods

This prospective observational study was conducted at the National Institute of Burn and Plastic Surgery (NIBPS), Dhaka, Bangladesh, between July 2022 and December 2023, following approval from the institutional ethics committee (SHNIBPS/ECC/2180). The study population comprised all patients admitted within 24 hours of sustaining burn injuries affecting 10-50% of the total body surface area (TBSA). Patients admitted more than 24 hours after injury, those with chronic infected wounds, and those unwilling to participate were excluded. Enrollment was carried out using purposive sampling.

The sample size was calculated using the single population proportion formula, n = Z²p(1−p)/d², based on a prevalence of bacterial growth in burn wounds of 68.5% reported by Chaudhary et al. [14], with a 95% confidence interval and 10% precision. The calculated sample size was 83, which was increased by 30% to account for potential non-response, yielding a final sample size of 110.

Demographic variables (age, sex), burn-related characteristics (type, depth, percentage, and site), and outcome measures (type of bacterial isolates, growth pattern, and antibiotic sensitivity) were recorded. Data were collected using a semi-structured questionnaire, patient interviews, and direct clinical observation.

Burn extent was assessed using the Lund and Browder chart [15]. Eligible patients were stabilized with standard burn management measures prior to sample collection. Written informed consent was obtained from patients or legal guardians after explaining the objectives, procedures, benefits, and risks of participation.

Wound swabs for culture and sensitivity were collected aseptically on the 1st, 5th, and 10th post-burn days. After donning sterile gloves, the wound bed was cleansed with sterile saline, and a sterile swab was rotated over approximately 1 cm² of viable tissue for several seconds. Dry wounds were pre-moistened with sterile saline before swabbing. Swabs were immediately placed in transport containers and delivered to the microbiology laboratory within one hour.

Specimens were inoculated onto blood agar and MacConkey agar. Bacterial identification was performed using colony morphology, Gram staining, and standard biochemical tests. Antimicrobial susceptibility testing was carried out on Mueller-Hinton agar using the Kirby-Bauer method of disc diffusion, following Clinical and Laboratory Standards Institute (CLSI) 2021 guidelines. Quality control was ensured using standard reference strains in accordance with CLSI recommendations.

Data were entered in Microsoft Excel and analyzed using IBM SPSS Statistics for Windows, Version 26 (Released 2018; IBM Corp., Armonk, New York, United States). Descriptive statistics were calculated for demographic and clinical variables, and results were presented as frequencies and percentages for categorical data and mean ± standard deviation for continuous variables. As repeated wound cultures were collected from the same patients at different time points, the McNemar test was used to compare paired categorical changes in bacterial growth status (growth vs no growth) between post-burn sampling days. A p-value < 0.05 was considered statistically significant.

Ethical clearance was obtained from the Institutional Review Board of SHNIBPS prior to study initiation, and permission was obtained from the hospital authority. Confidentiality was maintained by assigning identification numbers instead of names, and access to raw data was restricted to the principal investigator and the ethics committee. Participants were entitled to withdraw at any stage without affecting their care.

## Results

In Table 1, the largest proportion of patients was children under 10 years (43.6%), with a slight male predominance (56.4%). Flame burns were the most common etiology (50.0%), and the trunk was the most frequently affected site (73.6%). The mean TBSA burned was 22.9 ± 9.1%. 

**Table 1:** Demographic and Burn Characteristics of Participants (n=110) Values are expressed as frequency (percentage). Continuous variables are presented as mean ± standard deviation or median (range). **Multiple responses are allowed for the site of the burn. TBSA: total body surface area.

Variable	Category	n (%)
Age (in years)	<10	48 (43.6)
	10–20	22 (20.0)
	21–30	18 (16.4)
	31–40	11 (10.0)
	41–50	7 (6.4)
	51–60	3 (2.7)
	>60	1 (0.9)
	Mean ± SD	18.35 ± 16.1
	Median (Range)	14.5 (0.5–75)
Sex	Male	62 (56.4)
	Female	48 (43.6)
Type of burn	Flame	55 (50.0)
	Scald	26 (23.6)
	Electrical	23 (21.0)
	Chemical	1 (0.9)
	Others	5 (4.5)
Depth of burn	Superficial	22 (20.0)
	Superficial partial	29 (26.4)
	Deep partial	24 (21.8)
	Full thickness	12 (10.9)
	Mixed	23 (20.9)
Site of burn**	Trunk	81 (73.6)
	Upper limb	75 (68.2)
	Lower limb	63 (57.3)
	Neck	42 (38.2)
	Head	40 (36.4)
	Buttocks	27 (24.5)
	Perineum	6 (5.5)
% of TBSA burned	Mean ± SD	22.9 ± 9.1
	Median (Range)	20 (10–50)

**Figure 1:**
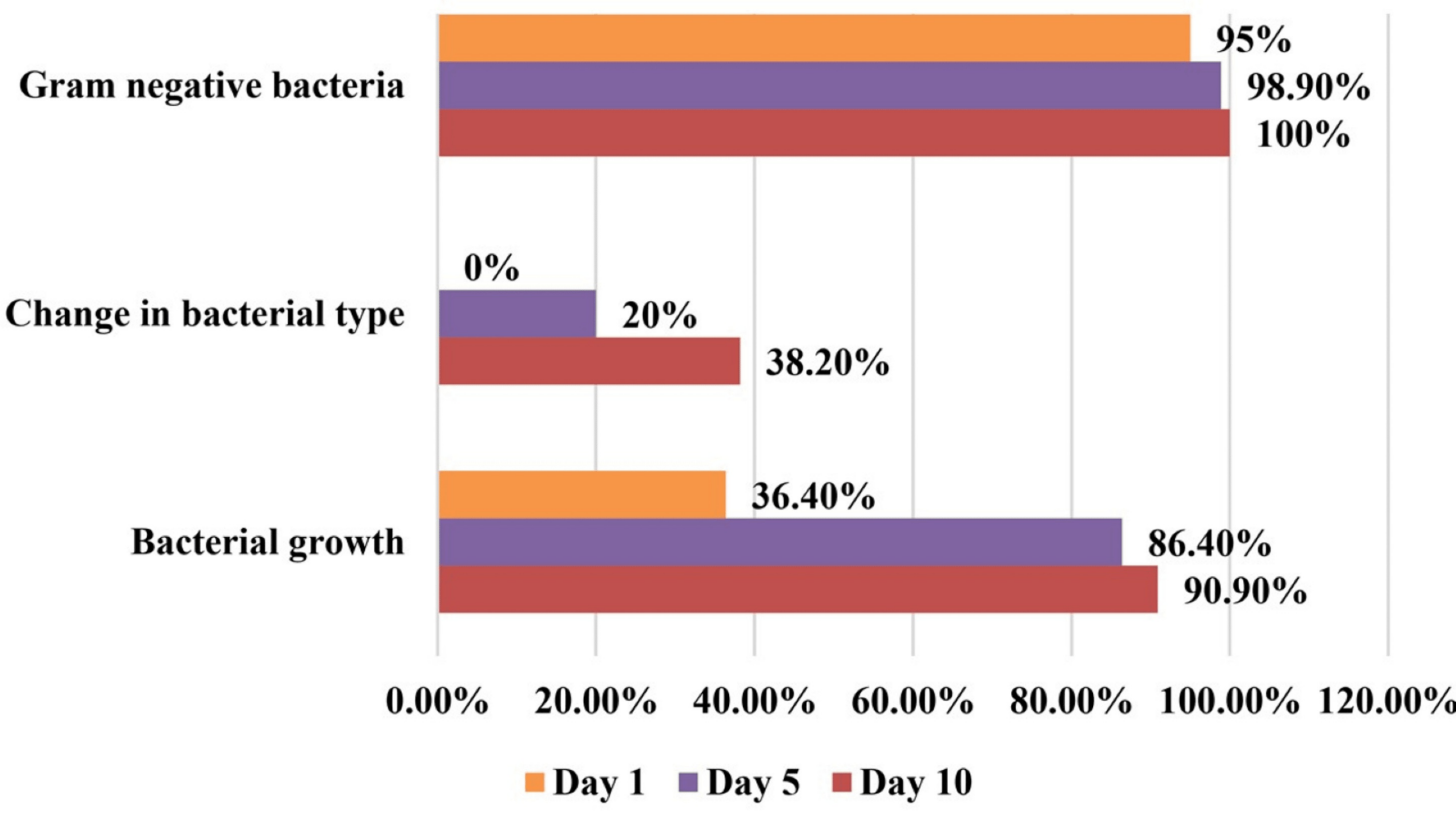
Trends in Bacterial Growth and Microbial Characteristics Over Time

In Figure 1, bacterial growth was observed in 36.4% of patients on day 1, of which 95% were gram-negative isolates. This proportion was 86.4% on day 5 and 90.9% on day 10. A change in bacterial type was observed in 20.0% of patients on day 5 and 38.2% on day 10.

In Table 2, colistin demonstrated the highest reported susceptibility against Pseudomonas isolates throughout the study period, although susceptibility declined from 76.9% on day 1 to 62.2% on day 10. Piperacillin-tazobactam showed moderate susceptibility, whereas meropenem, imipenem, aminoglycosides, and cephalosporins demonstrated comparatively lower susceptibility rates. Among Klebsiella isolates, colistin also showed comparatively higher susceptibility (65.5-80.0%), while gentamicin and cephalosporins exhibited consistently low susceptibility. Acinetobacter isolates demonstrated variable susceptibility patterns, with low susceptibility to most tested antibiotics across the study period.

**Table 2:** Antibiotic Sensitivity Patterns of Major Burn Wound Isolates n/N (%) indicates susceptible isolates/total isolates tested (%). Percentages were calculated using the total number of isolates for each organism as the denominator. The “other antibiotics” category included tigecycline, azithromycin, aztreonam, and ciprofloxacin.

Organism	Antibiotic	Day 1 n/N (%)	Day 5 n/N (%)	Day 10 n/N (%)
Pseudomonas	Colistin	20/26 (76.9%)	53/71 (74.6%)	56/90 (62.2%)
	Piperacillin–Tazobactam	14/26 (53.8%)	35/71 (49.3%)	40/90 (44.4%)
	Meropenem	3/26 (11.5%)	11/71 (15.5%)	8/90 (8.9%)
	Imipenem	6/26 (23.1%)	6/71 (8.5%)	12/90 (13.3%)
	Amikacin	6/26 (23.1%)	9/71 (12.7%)	9/90 (10.0%)
	Gentamicin	1/26 (3.8%)	2/71 (2.8%)	3/90 (3.3%)
	Cephalosporin	3/26 (11.5%)	5/71 (7.0%)	7/90 (7.8%)
	Other antibiotics	3/26 (11.5%)	17/71 (23.9%)	24/90 (26.7%)
Klebsiella	Colistin	8/10 (80.0%)	19/29 (65.5%)	18/27 (66.7%)
	Piperacillin–Tazobactam	3/10 (30.0%)	9/29 (31.0%)	9/27 (33.3%)
	Meropenem	3/10 (30.0%)	3/29 (10.3%)	4/27 (14.8%)
	Imipenem	1/10 (10.0%)	5/29 (17.2%)	5/27 (18.5%)
	Amikacin	2/10 (20.0%)	3/29 (10.3%)	5/27 (18.5%)
	Gentamicin	1/10 (10.0%)	1/29 (3.4%)	1/27 (3.7%)
	Cephalosporin	3/10 (30.0%)	3/29 (10.3%)	2/27 (7.4%)
	Other antibiotics	7/10 (70.0%)	7/29 (24.1%)	11/27 (40.7%)
Acinetobacter	Colistin	2/5 (40.0%)	6/9 (66.7%)	4/5 (80.0%)
	Piperacillin–Tazobactam	1/5 (20.0%)	5/9 (55.6%)	1/5 (20.0%)
	Meropenem	0/5 (0.0%)	3/9 (33.3%)	1/5 (20.0%)
	Imipenem	0/5 (0.0%)	2/9 (22.2%)	0/5 (0.0%)
	Amikacin	0/5 (0.0%)	2/9 (22.2%)	1/5 (20.0%)
	Gentamicin	0/5 (0.0%)	1/9 (11.1%)	0/5 (0.0%)
	Cephalosporin	0/5 (0.0%)	1/9 (11.1%)	0/5 (0.0%)
	Other antibiotics	5/5 (100.0%)	5/9 (55.6%)	2/5 (40.0%)

In Table 3, a significant increase in bacterial growth was observed between day 1 and day 5 (p < 0.001). No significant difference was observed between day 5 and day 10 (p=0.383).

**Table 3:** Comparing Bacterial Growth Over Time The McNemar test is applied to compare paired categorical variables over time. Only discordant pairs (no growth to growth and growth to no growth) are used for the calculation of p-values. The test statistics for Day 1 vs Day 5 were X² = 0.706, p < 0.001; X² = 0.378 for Day 5 vs Day 10, p = 0.383. *A p-value <0.05 is considered statistically significant.

Comparison	No Growth to Growth	Growth to No Growth	Continued Growth	Continued No Growth	p-value
Day 1 vs Day 5	59	4	36	11	<0.001*
Day 5 vs Day 10	13	8	87	2	0.383

## Discussion

In this study, most participants were children under 10 years (43.6%), with a mean age of 18.35 years (SD 16.1) and a median age of 14.5 years (range 0.5-75 years). This finding highlights the increased vulnerability of children to burn injuries, likely due to inadequate hazard awareness, close exposure to domestic cooking environments, and dependence on caregivers. A similar predominance of pediatric burn victims has been reported by Richcane and colleagues [16]. A slight male predominance (56.4%) was also observed, consistent with previous regional studies [14,17], possibly reflecting greater outdoor exposure and engagement in risk-prone activities among men.

Flame burns represented the most frequent etiology (50%), followed by scalds and electrical injuries. Similar findings were reported by Pednekar and others, where flame burns accounted for the majority of admissions [18]. The trunk and extremities were the most commonly affected sites, in agreement with previous reports [19]. Involvement of these large anatomical regions is clinically important because it increases fluid loss, metabolic stress, and susceptibility to invasive infection and sepsis.

Microbiological analysis demonstrated rapid wound colonization, with culture positivity increasing from 36.4% on day 1 to 86.4% on day 5 and remaining high on day 10. Gram-negative organisms predominated throughout the study period, with Pseudomonas aeruginosa as the leading isolate, followed by Klebsiella pneumoniae and Acinetobacter baumannii. Similar microbiological patterns have been reported in burn centers across Bangladesh and India [9,20]. Although early burn wound colonization is classically described as predominantly gram-positive, the marked gram-negative predominance observed even on day 1 in the present study may reflect local hospital flora, referral patterns, prior wound manipulation before admission, and early healthcare-associated microbial exposure in a tertiary burn center setting. The progressive predominance of gram-negative organisms during hospitalization may additionally reflect selective antimicrobial pressure, prolonged wound exposure, and persistent circulation of hospital-associated pathogens within burn units. Similar resistance trends have increasingly been reported across South Asian burn centers, raising concern regarding the emergence of multidrug-resistant organisms and progressively narrowing therapeutic options.

Antibiotic susceptibility testing revealed substantial resistance among the major gram-negative isolates, with no antibiotic demonstrating consistently high susceptibility throughout the study period. Although colistin demonstrated comparatively higher susceptibility than other tested antibiotics, susceptibility against Pseudomonas isolates declined from 76.9% on day 1 to 62.2% on day 10, indicating concerning resistance even to this last-line agent. Piperacillin-tazobactam showed moderate susceptibility, whereas meropenem, imipenem, aminoglycosides, and cephalosporins demonstrated comparatively lower susceptibility rates. Similar resistance patterns have been reported in South Asian burn centers, where therapeutic options against Pseudomonas and Acinetobacter species are becoming increasingly limited [21]. A recent genomic study from Bangladesh further demonstrated the presence of carbapenemase and β-lactamase genes among Pseudomonas isolates, supporting growing concern regarding antimicrobial resistance in burn units [22]. Increasing resistance to colistin reported in recent meta-analyses also raises concern regarding over-reliance on this antibiotic [23,24].

By day 10, over one-third of patients demonstrated polymicrobial colonization, consistent with findings by Chaudhary and colleagues [14]. Such polymicrobial growth may complicate empirical antimicrobial selection and contribute to delayed wound healing and secondary infection. However, clinical outcomes such as sepsis, graft failure, duration of hospitalization, and mortality were not systematically correlated with microbiological findings in the present study.

Overall, the findings highlight the potential importance of antimicrobial stewardship, periodic surveillance cultures, and burn-unit-specific antibiograms in guiding empirical antimicrobial selection in burn wound infections. Given the early predominance of Pseudomonas aeruginosa, institution-specific antibiograms and early protocol-guided empirical antibiotic selection may help reduce unnecessary use of last-line agents such as colistin in burn care settings. Future multicenter studies incorporating clinical outcomes such as sepsis, graft failure, duration of hospitalization, and mortality are recommended to better determine the clinical significance of burn wound colonization and antimicrobial resistance.

Limitations

This study has several limitations. First, it was conducted in a single tertiary burn center with a relatively small sample size, which may limit the generalizability of the findings. The use of purposive sampling may also have introduced selection bias and limited external validity. In addition, inclusion was restricted to patients with 10-50% TBSA burns, and therefore, the findings may not be generalizable to patients with more extensive or very severe burns. Second, superficial wound swab cultures were used instead of quantitative tissue biopsies; therefore, the microbiological findings may reflect surface colonization rather than true invasive burn wound infection. Third, fungal, anaerobic, and viral pathogens were not assessed, which limits comprehensive microbiological interpretation, particularly in burn patients exposed to prolonged hospitalization and broad-spectrum antibiotics. Fourth, molecular characterization of resistance mechanisms was not performed; therefore, statements regarding antimicrobial resistance were based only on phenotypic susceptibility patterns. Fifth, prior or concurrent antibiotic exposure, dressing protocols, and institutional antimicrobial prescribing practices were not systematically documented, although these factors may have influenced culture positivity and susceptibility patterns. Sixth, clinical outcomes such as sepsis, graft failure, duration of hospitalization, and mortality were not correlated with microbiological findings. Seventh, colistin susceptibility results should be interpreted cautiously because disc diffusion testing for colistin has recognized methodological limitations and is not the preferred method according to current CLSI/EUCAST recommendations. Finally, although serial cultures were obtained from the same patients, the statistical analysis was limited to descriptive comparisons and paired categorical testing, and more advanced longitudinal models were not applied.

## Conclusions

Burn wounds demonstrated rapid bacterial colonization during hospitalization, predominantly by gram-negative organisms, particularly Pseudomonas aeruginosa. Considerable resistance to commonly used antibiotics was observed, with no agent demonstrating consistently high susceptibility throughout the study period. These findings highlight the potential importance of periodic microbiological surveillance, burn-unit-specific antibiograms, and antimicrobial stewardship in guiding empirical antimicrobial selection in burn care settings. Further multicenter studies incorporating molecular resistance profiling and clinical outcome analysis are warranted.
